# Bayesian inference of local government audit outcomes

**DOI:** 10.1371/journal.pone.0261245

**Published:** 2021-12-14

**Authors:** Wilson Tsakane Mongwe, Rendani Mbuvha, Tshilidzi Marwala

**Affiliations:** 1 School of Electrical Engineering, University of Johannesburg, Auckland Park, South Africa; 2 School of Statistics and Actuarial Science, University of Witwatersrand, Johannesburg, South Africa; 3 Johannesburg Machine Intelligence Lab, Johannesburg, South Africa; Vellore Institute of Technology: VIT University, INDIA

## Abstract

The scandals in publicly listed companies have highlighted the large losses that can result from financial statement fraud and weak corporate governance. Machine learning techniques have been applied to automatically detect financial statement fraud with great success. This work presents the first application of a Bayesian inference approach to the problem of predicting the audit outcomes of financial statements of local government entities using financial ratios. Bayesian logistic regression (BLR) with automatic relevance determination (BLR-ARD) is applied to predict audit outcomes. The benefit of using BLR-ARD, instead of BLR without ARD, is that it allows one to automatically determine which input features are the most relevant for the task at hand, which is a critical aspect to consider when designing decision support systems. This work presents the first implementation of BLR-ARD trained with Separable Shadow Hamiltonian Hybrid Monte Carlo, No-U-Turn sampler, Metropolis Adjusted Langevin Algorithm and Metropolis-Hasting algorithms. Unlike the Gibbs sampling procedure that is typically employed in sampling from ARD models, in this work we jointly sample the parameters and the hyperparameters by putting a log normal prior on the hyperparameters. The analysis also shows that the repairs and maintenance as a percentage of total assets ratio, current ratio, debt to total operating revenue, net operating surplus margin and capital cost to total operating expenditure ratio are the important features when predicting local government audit outcomes using financial ratios. These results could be of use for auditors as focusing on these ratios could potentially speed up the detection of fraudulent behaviour in municipal entities, and improve the speed and quality of the overall audit.

## 1 Introduction

The Auditor General of South Africa (AG-SA) revealed that South African local government entities lost over $2 billion in irregular expenditure in the 2018-2019 financial year [1, 2]. This has consequently had a negative impact on service delivery and returns on the rapidly increasing government debt [1]. The manipulation of financial statements is not only limited to the public sector, with the Steinhoff collapse being a prime example of management fraud in the private sector [3]. Steinhoff is a South African retailer that lost over R200 billion in market capitalisation on the Johannesburg Stock Exchange (JSE) over a short space of time after allegations of accounting fraud [2, 3].

The recent scandal of Wirecard, and previously Enron, also indicate that financial statement fraud is not only a problem for South Africa, but the world at large [4, 5]. Wirecard is a German payment processing company, which filed for insolvency in 2020, that manipulated its financial statements by misstating its profit [4]. Enron was an American natural gas company that lost over $60 billion in market capitalisation in the early 2000s after the allegations of fraud emerged [5].

The use of automated techniques for the analysis and detection of financial statement fraud has been on the increase in the past decade [2, 6–13]. Mongwe and Malan [2, 6] outline how artificial intelligence and other automated methods can be used to construct decision supporters tools for various stakeholders. For example, auditors may use the decision support tool to flag entities who are at risks of having committed financial statement fraud and reduce the turn around time of audit amongst other benefits [2, 6]. A prime example within the South African context is the AG-SA having to audit all local and provincial government entities at the end of each financial year [1, 6].

Logistic regression has been successfully used in the literature for the detection of financial statement fraud [7, 14–21]. Moepya *et al*. [21] use logistic regression in the detection of fraud in companies listed on the JSE, while Boumediene *et al*. [22] performed a similar study for entities listed in Tunisia. Logistic regression has advantages over more complicated models such as artificial neural networks in that the results are more easily interpretable by the stakeholders, which is an important consideration when building a decision support tool [2, 6, 10, 21].

In logistic regression, as with any other machine learning model, one has to decide on the input features to use. Correctly selecting the variables to use as inputs for the models is important because it can influence the performance of the models [23]. Utilising feature selection techniques can improve the models predictive performance and reduce the model complexity as fewer features would be required [2, 24]. Examples of feature selection methods used in the financial statement fraud detection literature include correlation, t-test, analysis of variance, decision trees and principal component analysis [2, 21, 22, 25–28]. In this paper we limit ourselves to only using financial ratios for the prediction of financial statement audit outcomes. Thus feature selection in this context amounts to selecting which financial ratios are the most important or relevant in the inference of financial statement audit opinions.

In this work, we present the first use of Bayesian logistic regression with automatic relevance determination (BLR-ARD) for the inference of audit outcomes. The Bayesian approach allows us to measure the uncertainty in our predictions, which gives a sense of how much confidence we have in a particular prediction. The use of automatic relevance determination allows us to automatically determine which of the input features are the most relevant, with uncertainty measures around these as well [29]. This results in the model outcomes being more interpretable, allowing stakeholders to better understand the results of the model.

The motivation behind this work is in understanding the financial performance of South African municipalities in terms of audit outcomes, and particularly the features or financial ratios that drive these audit outcomes. We approach this problem from a Bayesian perspective, which is a first-in-literature, as it provides a probabilistically principled framework for predicting and understanding the audit performance of local government entities. This framework also enables us to provide uncertainty levels in the predictions produced by the models, and further allows us to automatically identify and rank the most important financial ratios for audit outcome modelling using prior distributions—which is an important contribution of this work.

The results of our analysis can be useful to various stakeholders, but particularly the Audit General of South Africa (AG-SA) and other auditors of local governments around the world. The results indicate which financial ratios the auditors could focus on so as to efficiently identify likely instances of financial irregular behavior and high financial risk in local government entities, and thus improve the speed and overall quality of the audit. As such audits are performed with limited resources—a framework such as the one presented in this work can be used for resource allocation on the basis of the predicted risk.

We train the BLR-ARD model parameters with Markov Chain Monte Carlo (MCMC) methods. MCMC methods have an advantage over approximate inference methods, such as variational inference and Laplace approximations, as MCMC methods are asymptotically guaranteed to converge to the true target posterior distribution [30]. In this paper, we present the first use of the Metropolis-Hasting (MH) algorithm, Metropolis Adjusted Langevin Algorithm (MALA), Separable Shadow Hamiltonian Hybrid Monte Carlo (S2HMC) and the No-U-Turn Sampler (NUTS) MCMC algorithms in the training of BLR-ARD models for inference of financial statement audit opinions.

The MH algorithm suffers from random walk behaviour [31–33]. This is due to the MH algorithm simply adding random Gaussian noise to the current state to determine the next state—with the noise being independent from the noise used to generate the current state [34]. The MH algorithm is often combined with other methods to construct hybrid Monte Carlo methods such as HMC [34–36].

The MALA improves on the MH algorithm by using first order gradient information. This reduces the random walk behaviour of the MH algorithm [36]. The MALA can be shown to be a special case of HMC with the trajectory length of one [35, 37]. As with the MALA, the HMC method uses first order gradient information of the target posterior to assist the exploration of the parameter space, but also adds an auxiliary momentum variable to the parameter space to allow it to explore different energy levels [32, 35, 38]. The HMC method has various parameters that need to be tuned, which is an impediment of the algorithm being broadly used in practice [39, 40]. The NUTS algorithm with dual averaging resolves this by adaptively setting the HMC parameters [39, 40].

The S2HMC algorithm has been shown to provide better sampling behaviour when compared to HMC [38, 41–43]. This is due to the shadow Hamiltonian in S2HMC being better conserved by the leap-frog integrator. This leads to higher acceptance rates and lower autocorrelations in the generated samples [38, 41, 44]. The main drawback of S2HMC is its high execution time, which reduces its performance on a execution time normalised effective sample size basis [38].

The empirical results in this work show that the S2HMC, NUTS and MALA algorithms are able to better explore the target posterior than the MH algorithm. This results in the S2HMC, NUTS and MALA algorithms having similar predictive performance, with the MH algorithm being significantly outperformed. The S2HMC method produces higher effective sample sizes than NUTS, indicating S2HMC’s ability to better explore the target posterior distribution than NUTS. The majority of the algorithms agree on which set of features are the most relevant for modeling audit opinions. The analysis shows that the current ratio, debt total revenue and the net surplus margin are the important features when predicting local government audit outcomes using financial ratios.

Note that multiple MCMC algorithms are considered in this work so as to assess the robustness of the results produced by the Bayesian framework. The results do indeed indicate that the methods agree on which financial ratios are important, suggesting that the Bayesian approach undertaken in this study is robust. Agreement on feature relevance from the multiple inference methods is key to avoiding spurious identification of relevant financial ratios.

The main contributions of this work are as follows:

- We present the first application of a Bayesian inference approach to the problem of predicting the audit outcomes of financial statements of local government entities using financial ratios.
- We present the first implementation of the BLR-ARD trained with the Separable Shadow Hamiltonian Hybrid Monte Carlo, No-U-Turn Sampler, Metropolis Adjusted Langevin Algorithm and Metropolis-Hasting algorithms.
- Unlike the Gibbs sampling procedure that is typically employed in Bayesian sampling from ARD models [29], in this work we jointly sample the model parameters and hyperparameters. This results in a more stable exploration of the posterior.

The remainder of this paper is structured as follows: Section 2 provides the background to financial statement fraud, Section 3 discuss the Markov Chain Monte Carlo methods used in this work, Section 4 outlines the experiments conducted and the dataset used, Section 5 presents and discusses the results of the experiments and we provide the conclusion in Section 6.

## 2 Overview of financial statement fraud detection

The financial statements of an entity consist of reports such as the income statement, balance sheet and cash flow statements [2, 6]. These statements are usually summarised into financial ratios, and used by different stakeholders for various purposes [2, 6]. For example, investors use the financial statements to determine if the entity is a good investment while the government would use financial statements to determine the tax payable to the state by the entity [2, 6]. These financial statement are typically summarised into financial ratios [2, 6].

Financial statement fraud, or management fraud, occurs when the financial statements of an entity are manipulated so as to make the entity appear to be in a better financial state than is actually the case, as was the case with Enron, Steinhoff and Wirecard [2, 4, 6, 45–47]. This manipulation is often perpetrated by the management of the entity, and at times with the support and knowledge of the auditors of the entity—as was the case with Enron [2, 5, 6, 47]. Examples of fraud that can be present in a financial statement of an entity include the omission of material information such as a large expense and the manipulation of the entity’s profits [2, 6, 13, 45].

In this paper, we model the audit opinion expressed by the AG-SA using financial ratios created from the local government entity’s financial statements. The audit opinion expressed by the AG-SA on the financial statements of South African municipalities falls broadly into the following categories [2, 6, 48]:

- *Clean or unqualified audit opinion*—The financial statements contain no material misstatements. Note that this does not necessarily mean there was no fraud.
- *Qualified audit opinion*—The financial statements contain material misstatements in specific amounts, or there is insufficient evidence to conclude that the amounts are not materially misstated.
- *Adverse audit opinion*—The financial statements contain material misstatements. This however does not necessarily mean that there was fraud present.
- *Disclaimer audit opinion*—The municipality provided insufficient evidence in the form of documentation on which to base an audit opinion.

From the above, it is clear that clean or unqualified audit opinion are preferred to the other audit opinions. For the purposes of this study, we consider the statements of a municipality to be a fraudulent instance if the audit opinion is not a clean or unqualified audit, which is consistent with other studies in the literature [2, 6, 12, 49]. Thus we consider a financial statement to be fraudulent if the AG-SA expressed a qualified, adverse or disclaimer audit opinion, and a financial statement is considered not fraudulent when it receives a clean or an unqualified audit opinion [2, 6].

## 3 Methodology

### 3.1 The model

In this work, we model the local government audit outcomes using Bayesian logistic regression. The negative log-likelihood *l*(D|**w**) function associated with logistic regression is given by:

$$\begin{array}{c} l\left(\text{D}|w\right)=\sum_{i}^{N}y_{i}\text{log}\left(w^{T}x_{i}\right)+\left(1-y_{i}\right)\text{log}\left(1-w^{T}x_{i}\right) \end{array}$$ (1)

where D is the data and *N* is the number of observations. Thus, the target unnormalised posterior log distribution is given as

$$\begin{array}{c} \text{ln}p(w|\text{D})=l(\text{D}|w)+\text{ln}p(w|\alpha )+\text{ln}q(\alpha ) \end{array}$$ (2)

where ln *p*(**w**|*α*) is the log of the prior distribution placed on the parameters given the hyperparameters, and ln *q*(*α*) is the distribution of the hyperparameters. We model the parameters **w** as having a Gaussian prior with each parameter having zero mean and its own standard deviation *α*_*i*_. The $\alpha_{i}^{\prime }s$ are assumed to follow a log-normal distribution with mean zero and variance 1. The *α*_*i*_ indicates how important the parameter associated with the input feature is. The larger the value of *α*_*i*_, the more important the input feature is in predicting the audit outcomes.

The aim is to infer the parameters **w** and hyperparameters *α* using Markov Chain Monte Carlo (MCMC) methods. In the literature, this problem is typically formulated as a Gibbs sampling scheme, where the hyperparameters are sampled first and then the parameters and so on [29, 31]. The approach taken in this paper is to jointly infer the parameters **w** and hyperparameters *α*. This approach has the advantage of resulting in a more stable exploration of the posterior, but results in the effective parameter space being doubled—which can significantly reduce the sampling time compared to the Gibbs sampling approach.

In the following sections we present the MCMC methods used to infer the posterior distribution in Eq (2).

### 3.2 Metropolis-Hastings algorithm and the Metropolis Adjusted Langevin Algorithm

The Metropolis-Hastings (MH) algorithm is one of the most basic MCMC methods. The MH algorithm generates the proposed samples using a user specified proposal distribution [32, 34]. The most commonly used proposal distribution in practice is the Gaussian distribution, with the mean being the current state, and the variance being a tunable parameter [32, 34]. This in-effect creates random walk behaviour as we are simply adding Gaussian noise to the current state, with the noise being independent from the noise used to generate the current state. This random walk behaviour results in very correlated samples, particularly in high dimensions [31]. In this work, we tune the variance parameter of the MH algorithm using dual averaging [39], targeting an acceptance rate of 70% during the burn-in period.

The Metropolis adjusted Langevin algorithm (MALA) is a MCMC sampler which aims to sample from the target distribution efficiently by using first order gradient information [36, 50]. Using the first order gradient information reduces the random walk behaviour associated with the MH algorithm [36]. The MALA uses Langevin dynamics to construct the Markov chain, with the dynamics given as [36, 50]:

$$\begin{array}{c} dw_{t}=\frac{1}{2}\nabla_{w}\text{ln}\pi \left(w\right)dt+dZ_{t} \end{array}$$ (3)

where *π*(**w**) represents the target probability density function, *w* represents the random variable that is to be sampled, *t* represents time, and *Z*_*t*_ is a Brownian motion process. Since this stochastic deferential equation is difficult to solve analytically, the first-order Euler-Maruyama discretisation is often used to provide an approximate solution, and the solution is written as [36, 50]:

$$\begin{array}{c} w_{t+1}=w_{t}+\frac{\epsilon^{2}}{2}\nabla_{w}\text{ln}\pi \left(w\right)+\epsilon z_{t} \end{array}$$ (4)

where *ϵ* is the step size and $z_{t}∼N\left(0,I\right)$.

The approximate solution introduces errors. In order to ensure detailed balance so that the generated chain converges to the target distribution, the Metropolis-Hasting acceptance-reject procedure is utilized. The transition probability of the MALA can be written as [50]:

$$\begin{array}{c} T\left(w^{\prime }|w\right)=N\left(\mu \left(w\right),\epsilon^{2}I\right), \end{array}$$ (5)

$$\begin{array}{c} T\left(w|w^{\prime }\right)=N\left(\mu \left(w^{\prime }\right),\epsilon^{2}I\right), \end{array}$$ (6)

$$\begin{array}{c} \mu \left(w^{\prime }\right)=w+\frac{\epsilon^{2}}{2}\nabla_{w}\text{ln}\pi \left(w\right), \end{array}$$ (7)

$$\begin{array}{c} \mu \left(w\right)=w^{\prime }+\frac{\epsilon^{2}}{2}\nabla_{w}\text{ln}\pi \left(w^{\prime }\right). \end{array}$$ (8)

where *T*(**w**′|**w**) and *T*(**w**|**w**′) are transition probability distributions, **w** is the last sample and **w**′ is the new sample generated. The final acceptance rate of the MALA takes the form:

$$\begin{array}{c} \text{min}\;[1,\frac{\pi \left(w^{\prime }\right)T\left(w^{\prime }|w\right)}{\pi \left(w\right)T\left(w|w^{\prime }\right)}] \end{array}$$ (9)

A key parameter that needs to be tuned for the MALA algorithm is the step size *ϵ*. We tune this parameter using primal dual averaging to target an acceptance rate of 70%.

Unlike the MH algorithm, the MALA takes advantage of the gradient information of the target distribution which makes the sampler converge to the target distribution more rapidly [36, 50]. However, the generated samples are still highly correlated. In the following section we present the Hamiltonian Monte Carlo and the No-U-Turn Sampler MCMC methods which use Hamiltonian dynamics for better exploration of the target distribution.

### 3.3 Hamiltonian Monte Carlo and the No-U-Turn Sampler

The Hamiltonian Monte Carlo (HMC) algorithm uses first order gradient information, in a similar fashion to MALA, of the target posterior to guide its exploration of the parameter space [37, 51]. However, unlike MALA, the HMC adds an auxiliary momentum variable **p** to the parameter space. The resultant Hamiltonian H(**w**, **p**) from this dynamic system is written as follows [31]:

$$\begin{array}{c} H(w,p)=U(w)+K(p) \end{array}$$ (10)

where U(**w**) is the negative log-likelihood of the target posterior distribution and K(**p**) is the kinetic energy defined by the kernel of a Gaussian with a mass matrix **M** [35]:

$$\begin{array}{c} K\left(p\right)=\frac{1}{2}\text{log}({\left(2\pi \right)}^{D}\left|M\right|)+\frac{p^{T}M^{-1}p}{2}. \end{array}$$ (11)

The trajectory of the Markov chain is driven by Hamilton’s equations at a fictitious time *t* as follows [31]:

$$\begin{array}{c} \frac{dw}{\partial t}=\frac{\partial H(w,p)}{\partial p};\;\frac{dp}{\partial t}=-\frac{\partial H(w,p)}{\partial w}. \end{array}$$ (12)

The evolution of this Hamiltonian system must preserve both volume and total energy. Furthermore, as the Hamiltonian is separable, to traverse the space we use the leapfrog integrator [31, 37]. In the leapfrog integrator, to reach the next point in the path, we take a half step in the momentum direction, followed by a full step in the direction of the model parameters and then ending with another half step in the momentum direction [23]. The update equations for the leapfrog integration scheme are [35, 37]:

$$\begin{array}{cc} p_{t+\frac{\epsilon }{2}} & =p_{t}+\frac{\epsilon }{2}\frac{\partial H(w_{t},p_{t})}{\partial w}\\ w_{t+\epsilon } & =w_{t}+\epsilon M^{-1}p_{t+\frac{\epsilon }{2}}\\ p_{t+\epsilon } & =p_{t+\frac{\epsilon }{2}}+\frac{\epsilon }{2}\frac{\partial H(w_{t+\epsilon },p_{t+\frac{\epsilon }{2}})}{\partial w}. \end{array}$$ (13)

Due to the discretisation errors arising from the leapfrog integration, a Metropolis-Hastings acceptance step is then performed in order to accept or reject the proposed sample [51], where the proposed sample parameters w* accepted with the probability [31]:

$$\begin{array}{c} \text{P}\left(\text{accept}\;w^{*}\right)=\text{min}\;(1,\frac{\text{exp}(-H(w^{*},p^{*}))}{\text{exp}(-H(w,p))}). \end{array}$$ (14)

The overall HMC sampling process utilises a Gibbs sampling scheme, where we sample the momentum and then sample a new set of parameters given the drawn momentum. Algorithm 1 shows the pseudo-code for the HMC where *ϵ* is a discretisation step size. The leapfrog steps are repeated until the maximum trajectory length *L* is reached.

**Algorithm 1**: Hamiltonian Monte Carlo

 **Input**: *N*, *ϵ*, *L*, *w*_init_, *H*(*w*, *p*)

 **Output**: ${\left(w\right)}_{m=0}^{N}$

1: *w*_0_ ← *w*_init_

2: **for** *m* → 1 **to** *N* **do**

3:  $p_{m-1}∼N\left(0,M\right)$

4:  *p*_*m*_, *w*_*m*_ = **Leapfrog**(*p*_*m*−1_, *w*_*m*−1_, *ϵ*, *L*, *H*)

5:  *δH* = *H*(*w*_*m*−1_, *p*_*m*−1_) − *H*(*w*_*m*_, *p*_*m*_)

6:  *α*_*m*_ = min(1, exp(*δH*))

7:  *u*_*m*_∼ Unif(0, 1)

8:  *w*_*m*_ = **Metropolis**(*α*_*m*_, *u*_*m*_, *w*_*m*_, *w*_*m*−1_)

9: **end for**

 **function** Leapfrog(*p*, *w*, *ϵ*, *L*, *H*)

10: **for** *t* ← 1 **to** *L* **do**

11:  $p\leftarrow p+\frac{\epsilon }{2}\frac{\partial H}{\partial w}(w,p)$

12:  *w* ← *w* + *ϵp*

13:  $p\leftarrow p+\frac{\epsilon }{2}\frac{\partial H}{\partial w}(w,p)$

14: **end for**

  **return** −*p*, *w*

  **function** Metropolis(*α*_*m*_, *u*_*m*_, *w*_*m*_, *w*_*m*−1_)

15: **if** *α*_*m*_ < *u*_*m*_ **then**

16:  *w*_*m*_ = *w*_*m*−1_

17: **else**

18:  *w*_*m*_ = *w*_*m*_

19: **end if**

  **return** *w*_*m*_

As shown in Algorithm 1, the HMC algorithm has multiple parameters that require tuning for efficient sampling, being the step size and the trajectory length. A trajectory length that is too short leads to a random walk behaviour similar to the Metropolis-Hasting method [32, 39]. A trajectory length that is too long results in a trajectory that inefficiently traces back [32, 39]. Similar conclusions can be drawn about the step size parameter. Tuning these parameters requires multiple time consuming pilot runs [32, 39].

The No-U-Turn Sampler (NUTS) automates the tuning of the leapfrog step size and trajectory length. In NUTS, the step size is tuned through primal dual averaging during the burn-in phase by targeting a specific sample acceptance rate [32, 39]. The trajectory length is tuned by iteratively doubling the trajectory length until either the chain starts to trace back or the Hamiltonian becomes infinite [32, 39, 40]. The empirical results have shown that NUTS performs at least as efficiently as and sometimes more efficiently than a well tuned standard HMC method, without requiring user intervention or costly tuning runs [39]. Thus, in this paper we use the NUTS algorithm, instead of the HMC, so that we do not perform any manual tuning of parameters.

### 3.4 Shadow Hamiltonian Monte Carlo

It can be shown that the leapfrog integrator only preserves the Hamiltonian up to second order [42, 44]. In order to increase accuracy, one could potentially design more accurate numerical integrators that preserve the Hamiltonian to a higher order, however, these approaches tend to be too computationally expensive [42]. Shadow Hamiltonians are perturbations of the Hamiltonian that are by design exactly conserved by the numerical integrator [38, 41], allowing one to determine the order as required.

The shadow Hamiltonian for a specific numerical integrator can be derived by performing backward error analysis on the integrator, with the shadow Hamiltonian being defined by an asymptotic expansion in the powers of the discretisation step size around the Hamiltonian [38]:

$$\begin{array}{c} \tilde{H}=H+\epsilon H_{2}+\epsilon^{2}H_{3}+\epsilon^{3}H_{4}+\ldots \end{array}$$ (15)

This asymptotic expansion diverges in practice, however a *k*^*th*^ order truncation of the expansion is used.

$$\begin{array}{c} \begin{array}{cc} {\tilde{H}}^{\left[k\right]} & =H+\epsilon H_{2}+\epsilon^{2}H_{3}+\epsilon^{3}H_{4}+\ldots \\ & =\tilde{H}+O(\epsilon^{k}) \end{array} \end{array}$$ (16)

The terms *H*_*k*_ can be determined by matching the corresponding components of the Taylor series in terms of *ϵ* and the expanded exact flow of the modified differential equation of the Hamiltonian [38]. These modified equations can be proved to be Hamiltonian for symplectic integrators such as the leapfrog [38].

In this work, we focus on a fourth-order truncation of the shadow Hamiltonian under the leapfrog integrator [38]. Since the leapfrog is second-order accurate ($O^{2}$), the fourth-order truncation is conserved with higher accuracy ($O^{4}$) than the true Hamiltonian [38]. The fourth-order shadow Hamiltonian for the leapfrog can be obtained by truncating the Baker–Campbell–Hausdorff (BCH) formula applied to Poisson brackets of the terms of the separable Hamiltonian [38, 41, 43, 52]:

$$\begin{array}{c} {\tilde{H}}^{\left[4\right]}=U\left(w\right)+K\left(p\right)+\frac{\epsilon^{2}}{12}K_{p}^{T}U_{\text{ww}}K_{p}-\frac{\epsilon^{2}}{24}U_{w}^{T}K_{\text{pp}}U_{w}+O\left(\epsilon^{4}\right) \end{array}$$ (17)

where *U*_**w**_, *U*_**ww**_, *K*_**p**_ and *K*_**pp**_ are Jacobians and Hessians of the potential and kinetic energies, respectively. The shadow Hamiltonian in Eq (17) is non-separable in terms of **w** and **p**, which necessitates computational expensive momenta acceptance criteria for momenta and potential tuning of additional parameters [38, 41, 43]. This additional computational overhead is overcome by pre-processing positions and momenta before propagating through the integrator [38, 41].

The Separable Shadow Hamiltonian Hybrid Monte Carlo (S2HMC) [41] algorithm utilises a processed leapfrog integrator to create a separable Hamiltonian. The separable Hamiltonian in S2HMC is:

$$\begin{array}{c} \tilde{H}\left(w,p\right)=U\left(w\right)+K\left(p\right)+\frac{\epsilon^{2}}{24}U_{w}^{T}M^{-1}U_{w}+O\left(\epsilon^{4}\right) \end{array}$$ (18)

Propagation of positions and momenta on this shadow Hamiltonian is performed after performing this reversible mapping $\left(\hat{w},\hat{p}\right)=X\left(w,p\right)$ where $\left(\hat{w},\hat{p}\right)$ through the following fixed point iterations [38, 41]:

$$\begin{array}{c} \begin{array}{cc} \hat{p} & =p-\frac{\epsilon }{24}(U_{w}\left(w+\epsilon M^{-1}\hat{p}\right)-U_{w}\left(w-\epsilon M^{-1}\hat{p}\right))\\ \hat{w} & =w+\frac{\epsilon^{2}M^{-1}}{24}(U_{w}\left(w+\epsilon M^{-1}\hat{p}\right)+U_{w}\left(w-\epsilon M^{-1}\hat{p}\right)). \end{array} \end{array}$$ (19)

After the leapfrog is performed this mapping is reversed using post-processing the following fixed point iterations [38]:

$$\begin{array}{c} \begin{array}{cc} w & =\hat{w}-\frac{\epsilon^{2}M^{-1}}{24}(U_{w}\left(w+\epsilon M^{-1}\hat{p}\right)+U_{w}\left(w-\epsilon M^{-1}\hat{p}\right))\\ p & =\hat{p}+\frac{\epsilon }{24}(U_{w}\left(w+\epsilon M^{-1}\hat{p}\right)-U_{w}\left(w-\epsilon M^{-1}\hat{p}\right)). \end{array} \end{array}$$ (20)

Once the samples are obtained from S2HMC as depicted in Algorithm 2, importance weights are calculated to allow for the use of the shadow canonical density rather than the true density [38, 41]. These weights are based on the differences between the true and shadow Hamiltonians as follows:

$$\begin{array}{c} b_{m}=\text{exp}(-\left(H\left(w,p\right)-\hat{H}\left(w^{\prime },p^{\prime }\right)\right) \end{array}$$ (21)

Mean estimates of observables *f*(**w**) which are functions of the parameters **w** can be computed as a weighted average [38, 41].

**Algorithm 2**: Separable Shadow Hamiltonian Hybrid Monte Carlo

 **Input**: *N*, *ϵ*, *L*, *w*_init_, *H*(*w*, *p*), $\hat{H}\left(w,p\right)$

 **Output**: ${\left(w\right)}_{m=0}^{N}$, importance weights = ${\left(b\right)}_{m=0}^{N}$

1: *w*_0_ ← *w*_init_

2: **for** *m* → 1 **to** *N* **do**

3:  $p_{m-1}∼N\left(0,M\right)$

4:  Apply the pre-processing mapping

$\left(\hat{w},\hat{p}\right)=X\left(w,p\right)$

5:  *p*_*m*_, *w*_*m*_ = **Leapfrog**(*p*_*m*−1_, *w*_*m*−1_, *ϵ*, *L*, $\hat{H}$)

6:  Apply the post-processing mapping $\left(w,p\right)=X^{-1}\left(\hat{w},\hat{p}\right)$

7:  $\delta H=\hat{H}\left(w_{m-1},p_{m-1}\right)-\hat{H}\left(w_{m},p_{m}\right)$

8:  *α*_*m*_ = min(1, exp(*δH*))

9:  *u*_*m*_∼ Unif(0, 1)

10:  *w*_*m*_ = **Metropolis**(*α*, *u*_*m*_, *w*_*m*_, *w*_*m*−1_)

11:  $b_{m}=\text{exp}\left(-\left(H\left(w_{m},p_{m}\right)-\hat{H}\left(w_{m},p_{m}\right)\right)\right)$

12: **end for**

In this paper, we set the trajectory length for the S2HMC algorithm to 100. We then tuned the step size using dual averaging [53], targeting an acceptance rate of 70% during the burn-in phase.

## 4 Experimental setup

In this section we, describe the dataset used and outline the experiments undertaken.

### 4.1 Data description

The raw dataset was obtained from the audited financial statement data of South African municipalities over the period of 2010 to 2018 [6]. The data was sourced from the South African National Treasury website [6, 54]: https://municipaldata.treasury.gov.za/, with the summarised version presented in Mongwe and Malan [6]. The dataset had a total of 1 560 records, of which 55% where non-fraudulent (i.e unqualified audit opinions). This shows that there is no large class imbalance in the data.

Table 1 provides descriptive statistics of the financial ratios, while Table 2 shows example financial ratio input features for three South African municipalities. The detailed construction of the ratios can be found in Mongwe and Malan [6], with the summary provided below:

1. *Debt to Community Wealth/Equity*—Ratio of debt to the community equity. The ratio is used to evaluate a municipality’s financial leverage.
2. *Capital Expenditure to Total Expenditure*—Ratio of capital expenditure to total expenditure.
3. *Impairment of PPE, IP and IA*—Impairment of Property, Plant and Equipment (PPE) and Investment Property (IP) and Intangible Assets (IA).
4. *Repairs and Maintenance as a percentage of PPE +IP*—The ratio measures the level of repairs and maintenance relative to assets.
5. *Debt to Total Operating Revenue*—The ratio indicates the level of total borrowings in relation to total operating revenue.
6. *Current Ratio*—The ratio is used to assess the municipality’s ability to pay back short-term commitments with short-term assets.
7. *Capital Cost to Total Operating Expenditure*—The ratio indicates the cost of servicing debt relative to overall expenditure.
8. *Net Operating Surplus Margin*—The ratio assesses the extent to which the entity generates operating surpluses.
9. *Remuneration to Total Operating Expenditure*—The ratio measures the extent of remuneration of the entity’s staff to total operating expenditure.
10. *Contracted Services to Total Operating Expenditure*—This ratio measures how much of total expenditure is spent on contracted services.
11. *Own Source Revenue to Total Operating Revenue*—The ratio measures the extent to which the municipality’s total capital expenditure is funded through internally generated funds and borrowings.
12. *Net Surplus / Deficit Water*—This ratio measures the extent to which the municipality generates surplus or deficit in rendering water service
13. *Net Surplus / Deficit Electricity*—This ratio measures the extent to which the municipality generates surplus or deficit in rendering electricity service.

**Table 1:** Five number summary of the 13 financial ratios. Note that mil represents million. Q1 and Q3 are the lower and upper quartiles. More information can be found in Mongwe and Malan [6].

Ratio	Min	Q1	Median	Q3	Max
1	-2046.45	11.97	19.43	35.49	6640.18
2	-3.81	11.22	17.85	25.36	77.66
3	-15.34	3.67	4.94	6.58	159.27
4	-1.86	0.00	0.83	1.91	170.67
5	-0.09	0.28	0.43	0.63	2.64
6	-0.88	0.56	1.16	2.19	23.06
7	-0.31	0.207	0.98	2.33	13.97
8	-147.32	-7.08	4.71	15.34	81.07
9	-67.61	26.31	32.51	41.17	159.98
10	-11.31	0.00	0.97	4.91	51.83
11	7.87	98.14	99.85	100.00	103.54
12	-114100 mil	0	0	83	1436 mil
13	-555400 mil	0	0	21	14970 mil

**Table 2:** Example of financial ratio input features for three South African municipalities in 2010. The BUF municipality had a qualified audit opinion while CPT and EKU has unqualified audit opinions.

Ratio	BUF Municipality	CPT Municipality	EKU Municipality
1	20.72	86.73	32.95
2	17.82	20.59	14.28
3	6.73	5.66	4.89
4	2.53	9.82	2.70
5	0.50	0.53	0.58
6	2.03	1.61	1.40
7	0.00	5.13	2.45
8	11.05	9.37	4.41
9	25.95	22.74	19.48
10	0.19	8.50	3.22
11	97.77	99.71	95.68
12	48.33	85.7	-7.62
13	28.93	31.12	25.51

### 4.2 Experiment description

For each of the MH, MALA, NUTS and S2HMC algorithms used in this paper, we generate five Markov chains of 10 000 samples. The first 5000 samples were used as the burn-in period, and any required tuning of algorithm parameters was performed during the burn-in period. For the NUTS and S2HMC algorithms we set the pre-conditioning matrix **M** = **I**, which is the common approach in practice [31, 33, 38].

We then assess the performance of the algorithms by generating the trace plots of the unnormalised target posterior distribution, the effective sample sizes of the generated samples, the effective sample sizes of the generated samples normalised by execution time as well as predictive performance on unseen data. Note that the execution time is the time taken to generate the samples after the burn-in period.

The ESS calculation used in this paper is the multivariate ESS metric outlined in Vats *et al*. [38, 55]. Unlike the minimum univariate ESS measure typically used to analyse MCMC results, the multivariate ESS measure of Vats *et al*. [38, 55] takes into account the correlations between the different parameter dimensions [36, 38, 55]. The minimum univariate ESS metric has the disadvantage that the estimate of the ESS ends up being dominated by the parameter dimensions that mix the slowest [38, 55]. For the S2HMC algorithm, which is an importance sampler, the multivariate ESS is adjusted by taking into account the possibility of non-uniform importance weights ${\left(b\right)}_{m=0}^{N}$ through the thinning algorithm outlined in Radivojevic *et al*. [38, 42].

The predictive performance on unseen data is performed using the accuracy measure, the receiver operating curve (ROC) as well as area under the curve (AUC). ROC plots the true positives from the model on the y-axis against the false positives on the x-axis. AUC is the area under the ROC, and represents the average miss-classifications rate. AUC is useful as a performance measure when the costs of classification are unknown, which is the case for the financial statement fraud domain [2, 21, 56, 57].

The ranking of the importance of the financial ratios is performed by calculating the mean or average *α*, which are the standard deviations in Eq (2), for each model parameter over the five chains. The higher the *α* value, the more important the input financial ratio is to the modelling of the audit outcomes.

## 5 Results and discussion

The experiments were implemented in PyTorch and were carried out on a 64-bit precision CPU. In evaluating the S2HMC algorithm, we set a convergence tolerance of 10^−6^ or the completion of 100 fixed point iterations.

**Fig 1:**
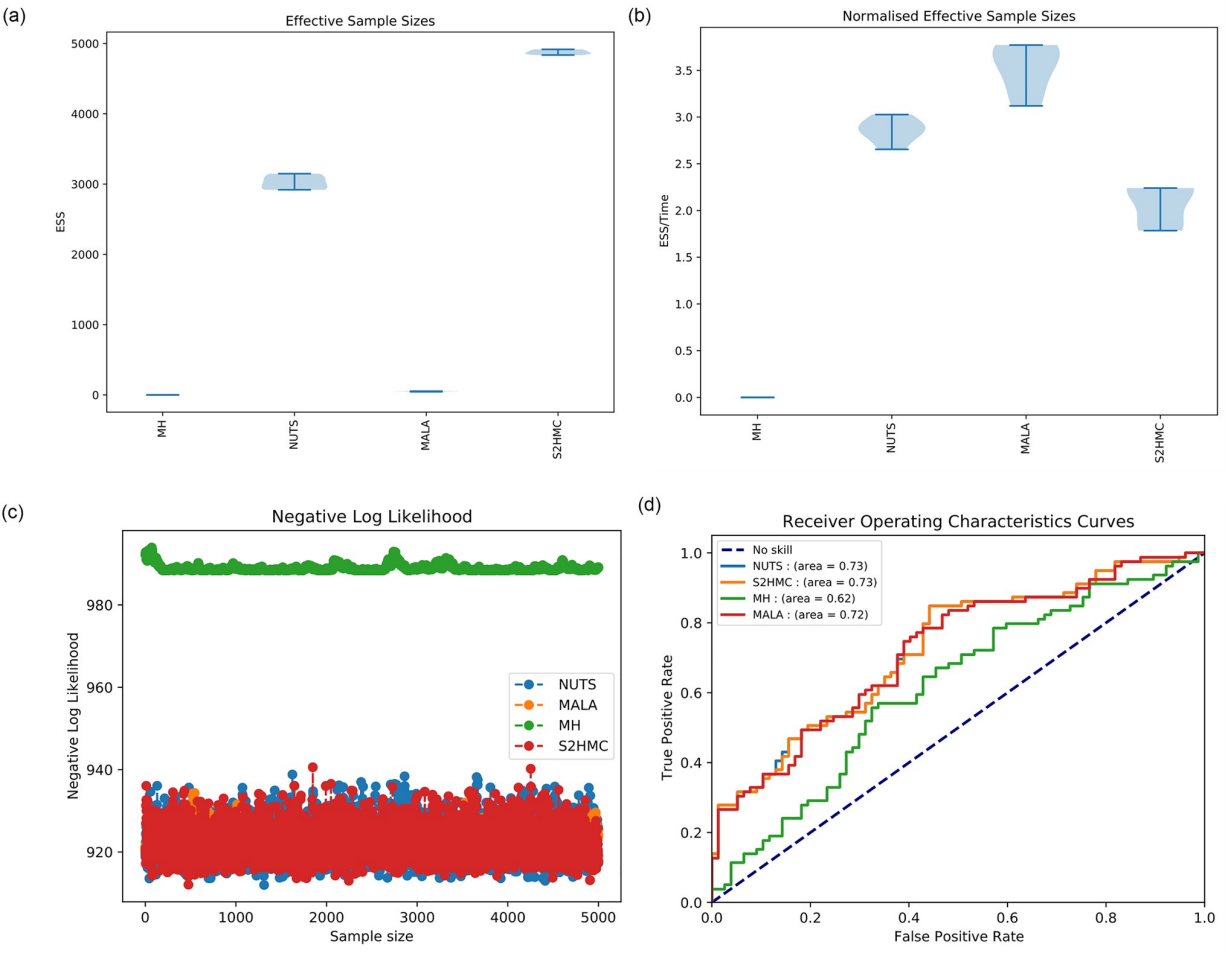
Inference results for the BLR-ARD model across various sampling methods. a) Effective sample sizes, b) Effective sample sizes normalised by execution time, c) Diagnostic negative log-likelihood trace plots and d) Predictive performance based on the Area under the Receiver Operating Curve.

**Fig 2:**
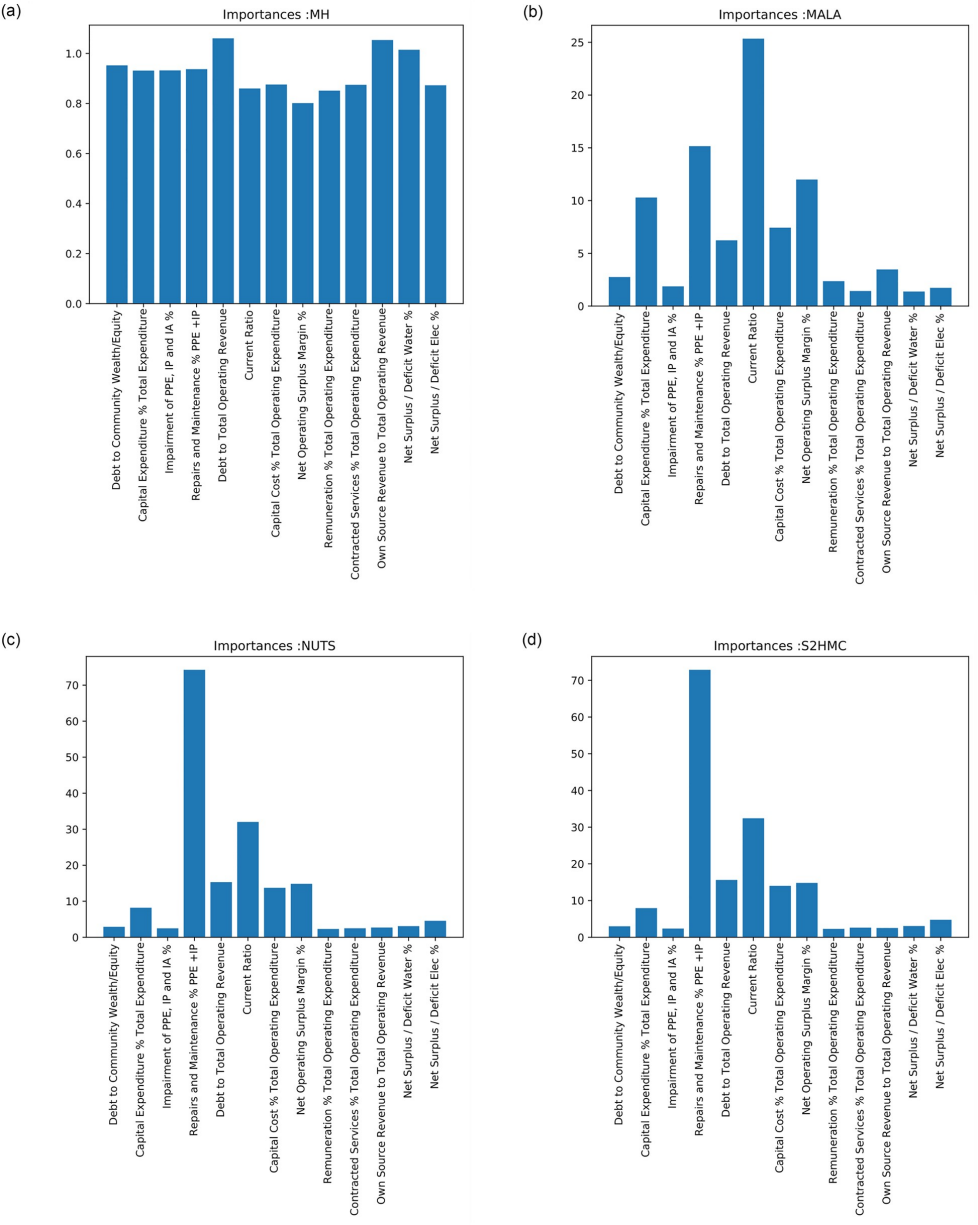
Mean posterior variances from each of the algorithms. The higher the value, the more important the financial ratio is to the task of modelling audit opinions. a) Importance’s for MH, b) Importance’s for MALA, c) Importance’s for NUTS and d) Importance’s for S2HMC.

Fig 1 shows the inference results, while Fig 2 shows the input feature importance or relevance. The results of Fig 2 are summarised, as rankings for each financial ratio, in Table 4. Table 3 shows the predictive performance of the samplers based on the Area under the Curve (AUC) and accuracy performance metrics respectively.

**Table 3:** Area under the receiver operating curve (AUC) and accuracy of the MCMC methods. The results were averaged over 10 runs of each algorithm.

Metric	MH	MALA	NUTS	S2HMC
AUC	0.624	0.723	0.732	0.733
Accuracy	0.651	0.737	0.744	0.753

**Table 4:** Ranking of the financial ratios by each method. For example, NUTS ranks ratio 4 as the most important, while MH ranks ratio 12 as the third most important.

Ranking	MH	MALA	NUTS	S2HMC
1	5	6	4	4
2	11	4	6	6
3	12	8	5	5
4	1	2	8	8
5	4	7	7	7
6	3	5	2	2
7	2	11	13	13
8	7	1	12	12
9	10	9	1	1
10	13	3	11	10
11	6	13	10	11
12	9	10	3	3
13	8	12	9	9

Fig 1(a) shows that the S2HMC produces the highest effective sampling sizes, indicating that the algorithm produces less correlated samples when compared to the other methods. NUTS has the second highest effective sample sizes, with MH and MALA having very low effective sample sizes, indicating that these two methods produce very correlated samples. However, Fig 1(b) shows that on a normalised (by execution time) effective sample size basis, the MALA outperforms all the methods. This is testament to the fact that MALA has a very low execution time compared to NUTS and S2HMC. Although MH is also relatively fast, since the effective sample size it produces is zero, it still underperforms on a normalised effective sample size basis.

Fig 1(c) shows that the MH algorithm converges to a higher, and different negative log-likelihood than the other methods. This highlights the very poor exploration capabilities of the MH algorithm. Fig 1(d) shows that the MH algorithm has the lowest predictive performance. S2HMC and NUTS have the joint highest predictive performance, which corresponds with the high effective sample sizes generated by these methods.

Fig 2 shows the relative importance or relevance of each of the financial ratios produced by each of the MCMC methods. The results show that the MH algorithm struggles to distinguish between important and not-so-important financial ratios. This is because of the poor exploration of the target. On the other hand, the other three methods are able to extract the importance or most relevant features for the audit opinion modeling task. Table 4 shows the ranking of the importance of the financial ratios produced by each of the method. The most commonly featured financial ratios in the top five ranking are:

- Ratio 4—*Repairs and Maintenance as a percentage of PPE +IP*: This ratio is selected by all four methods
- Ratio 5—*Debt to Total Operating Revenue*: This ratio is selected by the MH, NUTS and S2HMC methods
- Ratio 6—*Current Ratio*: This ratio is selected by the MALA, NUTS and S2HMC algorithms
- Ratio 8—*Net Operating Surplus Margin*: This ratio is selected by the MALA, NUTS and S2HMC algorithms
- Ratio 7—*Capital Cost to Total Operating Expenditure*: This ratio is selected by the MALA, NUTS and S2HMC algorithms.

These results are inline with those observed by Mongwe and Malan [6] for local government entities. Mongwe and Malan [6] used self-organising maps and found that the financial ratios associated with fraudulent financial statements are the current ratio, net operating surplus margin and the debt to total operating revenue. In this work, our analysis shows that the most relevant financial ratio is the repairs and maintenance as a percentage of PPE and IP, followed by the ratios found in Mongwe and Malan [6], with the fifth most relevant ratio is the capital cost to total operating expenditure ratio.

These results make intuitive sense as, for example, a high repairs and maintenance ratio means that the municipality is doing more repairs to assets than the total value of assets that it has, which is likely an indication of lack adherence to proper corporate governance as repairs to assets should typically be less than the value of those assets—else those assets should be written-off. Furthermore, a high capital cost to total expenditure ratio means that debt repayments are the largest component of total expenditure, indicating that the entity has a large amount of debt—which might prompt the entity to act in a manner that flouts corporate governance procedures in order to hide its dire financial situation.

Mongwe and Malan [6] provide an interpretation of the current ratio, net operating surplus margin and capital cost to total operating expenditure financial ratios in terms of how they relate with audit outcomes. Our findings agree with Mongwe and Malan [6] in that we find that a high current ratio, a high net surplus operating margin and low debt to total operating revenue financial ratios are associated with entities that are less likely to engage in manipulation of their financial statements as they are in good financial standing—with the converse also being true.

These results can prove to be particularly useful for auditors as focusing on these ratios can speed up the detection of inadequate corporate governance behaviour in municipal entities, and improve the overall quality of the audits.

## 6 Conclusion

We present the first fully Bayesian approach to the inference of financial statement audit opinions. This Bayesian approach is applied to local government entity audit outcomes using financial ratios as inputs. The inference is performed using Metropolis-Hastings, Metropolis Adjusted Lengavin Algorithm, No-U-Turn Sampler and Separable Shadow Hybrid Hamiltonian Monte Carlo algorithms. The sampling was applied to Bayesian Logistic Regression with automatic relevance determination. Automatic relevance determination (ARD) allows one to determine which features are the most important in an automated manner, and thus performing feature selection in an implicit fashion.

In this work, the parameters and the hyperparameters, which measure the relevance of the financial ratios, are jointly sampled. The results show that the Separable Shadow Hybrid Hamiltonian Monte Carlo produces the best sampling results, with the highest effective sample sizes. However, the predictive performance of the No-U-Turn Sampler and Separable Shadow Hybrid Hamiltonian Monte Carlo algorithms is found to be the same. The Metropolis-Hasting algorithm produces the worst sampling behaviour due to its random walk nature, and has both the lowest effective sample rates and predictive performance.

The results further show that the most important features in the modelling of audit outcomes for municipalities are the repairs and maintenance as a percentage of total assets ratio, current ratio, debt to total operating revenue, net operating surplus margin and capital cost to total operating expenditure ratio. This could prove to be useful for auditors as focusing on these ratios can speed up the detection of possible fraudulent behaviour of municipal entities.

This work can be improved upon by comparing the performance of the Bayesian Logistic Regression with ARD model with other models such as the Bayesian Neural Network with ARD model. Furthermore, we plan on performing this analysis for listed entities in addition to the local government entities considered in this work. The consideration of a larger set of financial ratios could also improve the results. In addition, Riemannian manifold based Markov Chain Monte Carlo methods could also be considered as they are able to better explore the target posterior distribution due to their ability to take into account the local geometry of the target distribution.
